# Short-term effects of national smoking cessation service on smoking-related disease prevalence and healthcare costs: Experience from the National Health Insurance Service Smoking Cessation Intervention Program in Korea

**DOI:** 10.18332/tid/169654

**Published:** 2023-08-24

**Authors:** Jin-Kyoung Oh, Minji Han, Min Kyung Lim

**Affiliations:** 1 Department of Cancer Control and Population Health, National Cancer Center Graduate School of Cancer Science and Policy, Goyang, Republic of Korea; 2 Department of Health Science and Technology, Graduate School of Convergence Science and Technology, Seoul National University, Seoul, Republic of Korea; 3 Department of Social and Preventive Medicine, College of Medicine, Inha University, Incheon, Republic of Korea

**Keywords:** smoking, cessation, benefit, cost, Korea

## Abstract

**INTRODUCTION:**

We measured the short-term clinical and economic impacts of the National Health Insurance Service (NHIS) smoking cessation program, which subsidizes the cost of pharmacotherapy and medical consultations, by comparing the changes in prevalence and healthcare costs of smoking-related diseases among cessation service users, non-users, and never smokers.

**METHODS:**

Smokers who used the cessation service from 2015 to 2017 were included (n=779315). We used claims data from the NHIS, a mandatory, single-payer insurance covering the entire Korean population, to determine the number of patients with selected diseases, their healthcare utilization, and medical costs, and compared these amounts in the one year before and after enrollment. For further comparison, we also estimated disease prevalence and medical costs in matched controls by age, sex, income, and residential area, including never smokers and smokers who never used the cessation program.

**RESULTS:**

Across all 15 selected diseases, the number of patients, days spent in the hospital, and medical costs for 1 year were consistently higher after service enrollment than before. This pattern was observed for both men and women. Notably, decreased prevalence and medical costs for pneumonia were observed among individuals aged <50 years. Healthcare utilization for any kind of disease for 1 year was 97.7%, 91.1%, and 88.8% among cessation service users, never smokers, and smokers who did not use the cessation service, respectively. The disease-specific prevalence was also highest and increased more in the cessation service users compared with the control groups.

**CONCLUSIONS:**

Cessation service users were more likely to seek healthcare. Increased healthcare utilization in the first year after cessation service use may have resulted from smoking-related conditions that led individuals to attempt smoking cessation.

## INTRODUCTION

Smoking cessation treatment is considered crucial for tobacco users as it helps prevent chronic diseases and premature deaths causally linked to tobacco use^1^. The health consequences of tobacco use include cancers, stroke, diabetes, and other chronic diseases, as well as more general adverse effects on the body, such as inflammation and impairment of immune function^2^. The short-term clinical outcomes of tobacco cessation include a lower risk of death from respiratory diseases and cancer^3^, a lower risk of secondary coronary heart disease events and death^4,5^, and a lower rate of diabetes^6^.

Article 14 of the World Health Organization’s (WHO) Framework Convention on Tobacco Control (FCTC) recommends the implementation of tobacco cessation interventions – brief advice in primary healthcare settings, national toll-free quitlines, and pharmacotherapy, including at least nicotine replacement therapy (NRT) – as an important component of comprehensive tobacco control programs^7,8^. Many countries have offered a combination of these interventions as the most effective method of helping people quit smoking^9-11^. Following the WHO recommendations, population-based approaches, such as quitlines, the internet, and self-help interventions, have been successfully implemented in countries where governments can invest in promoting those services^7^. Healthcare providers in the existing healthcare system have also been encouraged by the government to be on the frontline of tobacco cessation for more consistent and effective identification and treatment of tobacco users. However, as the existing healthcare systems are not sufficiently organized to address the issue of tobacco use, such attempts were not very successful even in high-income countries, and there is a marked lack of coverage of the tobacco control measure on ‘offering cessation’ in MPOWER (32% of the world population in 2018)^8^.

In Korea, the successful implementation of population-based cessation approaches, e.g. public health center-based cessation services (since 2005), a national toll-free quitline (since 2006), and internet-based cessation services (since 2013), combined with the enforcement of other tobacco control measures, significantly reduced the smoking rate among men from 51.6% in 2001 to 39.3% in 2015^12^. However, the prevalence of smoking remains high, and the economic burden attributable to smoking is also significant, as illustrated by the direct medical costs of cancer attributable to smoking: US$531 million in 2014, or 1.2% of the total Korean national healthcare expenditure for that year^13^. With the tobacco tax increase in 2015, the National Health Insurance Service (NHIS) started to cover the cost of tobacco cessation consultation and drug fees in hospitals and clinics across the country. The service introduced systematic changes to the screening and documentation of tobacco use in patient health records; implemented training for healthcare providers on effective, evidence-based cessation support; defined rules for including cessation support in routine care; and developed a budget and resources for delivering effective cessation support. The cessation service currently covers approximately 300000 smokers who visit clinics and hospitals for medical care or health check-ups and decide to quit by the physicians’ brief advice, annually^14^.

In this study, to see the effect of cessation service use, we measured changes in the prevalence and related medical costs of selected diseases among cessation service users 1 year before and after the service enrollment, which have never been reported, and compared these amounts with those of never smokers and smokers who did not use the service.

## METHODS

### Data

*National Health Insurance Service smoking cessation intervention data*

The NHIS smoking cessation program in clinics and hospitals was launched across Korea in 2015, based on strong evidence that behavioral support and pharmacotherapies (i.e. varenicline, bupropion, and NRT) improve individuals’ chances of successfully quitting smoking^9,10^. Currently, approximately 20% of clinics and hospitals in Korea have joined the program, following an increasing trend. The program subsidizes doctor consultation fees for up to six appointments and supports the purchase of smoking cessation medications (i.e. bupropion, varenicline, and NRT) for 8 to 12 weeks once registered. A total of three quit attempts using the program are covered by NHIS per year regarding cases that relapse. The program offers a gift for healthcare valued at US$80 as an incentive to complete the 12-week schedule, proven by attending six hospital/clinics visits for consultation or receiving a prescription for cessation medication from 8 to 12 weeks^14,15^. Furthermore, the program offers each participant a referral for the quitline’s additional cessation maintenance services after completion of the program, and their baseline and contact information are delivered to the quitline with the participant’s consent^15^. Each service user’s baseline characteristics, including smoking behavior, history of service use, and cessation outcomes, are recorded in the electronic system managed by the National Health Insurance Cooperation (NHIC)^15^. Healthcare providers in various settings, including all public or private clinics and hospitals, as well as all medical departments, can register for service delivery and are trained via a structured education program that is available both offline and online, and consists of seven classes of approximately 4 hours each^16^. By 2018, 1.1 million smokers (i.e. approximately 12% of the 9.5 million adult smokers in Korea) had used the NHIS cessation service at least once (internal data).

*Health insurance claims data*

This study used health insurance claims data provided by the NHIC. The NHIS is a single-payer mandatory insurance that provides benefits for the prevention, diagnosis, and treatment of disease and injury as well as for rehabilitation, births, deaths, and health promotion. All South Korean citizens must either be enrolled in the NHIS (97% of the entire population) or otherwise receive medical aid (3%)^17^. The NHIS database contains information on medical aid subjects for the entire Korean population. Currently, the NHIS maintains and stores national records for healthcare utilization and prescriptions. The NHIS claims data include the cost of care, medical institution attended, income level, and residence of all insurance subscribers^18^. These claims data are linked with the national health screening database, which provides self-reported health behaviors (e.g. smoking, alcohol consumption) and clinical examination results (e.g. body mass index [BMI], fasting blood sugar [FBS], and total cholesterol [TC]). All insured adults are eligible for the biennial NHIS general health screening program^18^ for which the participation rate among the eligible population in 2014 was 74.8%^17^.

### Study design and population

**Figure 1:**
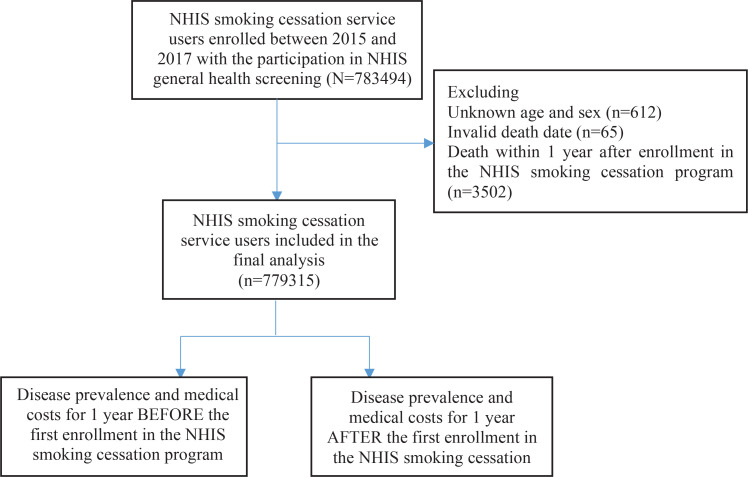
Flow chart for selection of study population for comparing the diseases prevalence and the medical costs before and after enrollment in the National Health Insurance Service (NHIS) smoking cessation program

**Figure 2:**
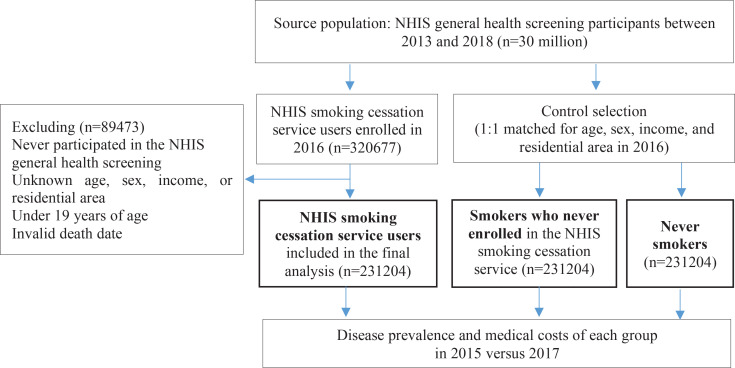
Selection of population in cases (National Health Insurance Service (NHIS) smoking cessation service users) and controls (smokers who did not use the service and never-smokers) for comparing the diseases prevalence and the medical costs between them

In the current study, we obtained two analytical samples from the NHIS database. One is for comparison between pre and post of smoking related diseases prevalence and their medical cost among users of the NHIS smoking cessation intervention program. Smokers who used the NHIS smoking cessation intervention program from 2015 to 2017 and participated in the NHIS general health screening were included in the study as the baseline population (n=783494). After excluding individuals with unknown age or gender (n=612), invalid date of death (n=65), or death within 1 year after enrollment of the service (n=3502), we included 779315 service users in the first analytical data to evaluate changes in disease prevalence and related medical costs, before and after the calendar year when NHIS smoking cessation intervention program was used (Figure 1). The other is for comparison of smoking-related diseases prevalence and their medical cost between cases (smokers who used the cessation program) and controls (smokers who did not use the cessation program and lifelong never smokers). From the 30 million individuals in the NHIS general health screening participants between 2013 and 2018, a subgroup of NHIS smoking cessation intervention program users in 2016 (n=320667) was extracted. After excluding 89473 individuals who were never screened, those with unknown matching variables (i.e. age, sex, income level, and residential area), those aged <19 years, and those with an invalid death date, we finally included 231204 users as cases in the second analytical data. The same number of current smokers (n=231204) and never smokers (n=231204) were selected after frequency matching for age, sex, income level, and residential area (Figure 2). Under internal regulation of NHIS to minimize the amount of data released regarding privacy protection and analytical eligibility, the NHIS cessation service users in 2016 were only included as cases in the second analytical data to reduce the huge amount of control data added from NHIS general health screening participants in 2013–2018.

### Baseline characteristics

We collected the baseline characteristics of service users, including gender, age, household income (quintiles), residential area (urban, rural), number of cessation service enrollments (<1, 1–2, ≥3), 6-month smoking cessation outcome (success, failure), BMI (<18.5, 18.5–23, 23–25, ≥25 kg/m^2^), FBS (<100, 100–126, ≥126 mg/dL), TC (<200, 200–240, ≥240 mg/dL), and alcohol consumption per week (<1, 1–2, 3–4, ≥5 times).

### Disease prevalence and medical costs

We used the NHIS claims data to determine the number of patients and events in the healthcare sector and estimate the disease prevalence and medical costs associated with inpatient and outpatient care. Diseases known to be associated with active smoking were selected based on the following International Classification of Diseases 10th Revision (ICD-10) primary disease codes: tuberculosis (A15–19), cancer (C00–97), diabetes mellitus (E10–14), cataracts (H25–28), ischemic heart diseases (I20–25), cerebrovascular diseases (I60–69), diseases of the arteries, arterioles, and capillaries (I70–79), acute upper respiratory infections (J00–06), influenza (J09–11), pneumonia (J12–18), other acute lower respiratory infections (J20–22), other upper respiratory tract diseases (J30–39), chronic lower respiratory diseases (J40–47), rheumatoid arthritis (M05–06), and impotence (N48.4). NHIS claims data included all of the actual costs for medical care for each individual. From the sum of all specific medical diagnoses, the comprehensive medical cost used for every disease of each individual was estimated. We compared the changes in disease prevalence (point prevalence of each year observed and compared), days spent in the hospital, and medical costs in 2015 and 2017 for each group. We estimated the medical costs of smoking-associated diseases from an expanded healthcare payer’s perspective, which included patient co-payments and service and practice costs (e.g. for doctors’ consultations, injections, operations, diagnostic testing/imaging, prescriptions, and bed usage).

## RESULTS

Table 1 shows the general characteristics of the study population. The NHIS smoking cessation service users were predominantly men (86.5%) and aged ≥40 years (74.3%). Half of the users (49.1%) were in the upper middle or high-income quintiles. Seventy-three percent of smokers enrolled in the service once, and 27% enrolled multiple times. Of all the service users, 35.2% completed the full cessation program by attending six hospital/clinic visits or receiving a prescription for 8–12 weeks. About one-third (34.8%) were obese (i.e. BMI ≥25 kg/m^2^), 9.7% had high blood sugar (FBS ≥126 mg/dL), 10.7% had high TC (≥240 mg/dL), and 68.7% drank alcohol at least once per week.

**Table 1:** General characteristics of the study populations: National Health Insurance Service (NHIS) smoking cessation service users enrolled between 2015 and 2017 (N=779315)

*Characteristics*	*n*	*%*
**Sex**		
Men	673967	86.5
Women	105348	13.5
**Age** (years)		
<19	2728	0.4
19–29	49495	6.4
30–39	147674	18.9
40–49	230801	29.6
50–59	205898	26.4
60–69	108717	14.0
≥70	34002	4.4
**Household income^a^**		
Lowest (medical aid recipient)	33381	4.4
Low (1st quintile)	101720	13.3
Lower middle (2nd quintile)	110609	14.4
Middle (3rd quintile)	144138	18.8
Upper middle (4th quintile)	180120	23.5
High (5th quintile)	195818	25.6
**Residential area**		
Urban	337106	43.3
Rural	442209	56.7
**Number of deaths^b^**	9832	1.3
**Number of cessation service enrollments**		
1	569003	73.0
2	142294	18.3
≥3	68018	8.7
**Outcome of cessation service use^c^**		
Success^d^	274351	35.2
Failure	504964	64.8
**Body mass index** (kg/m^2^)^a^		
<18.5	13672	1.8
18.5–23	176332	22.6
23–25	155147	19.9
≥25	270978	34.8
**Fasting blood sugar** (mg/dL)^a^		
<100	334441	42.9
100–126	205985	26.4
≥126	75837	9.7
**Total cholesterol** (mg/dL)^a^		
<200	338762	43.5
200–240	193952	24.9
≥240	83545	10.7
**Alcohol consumption** (times/week)^a^		
0	192660	31.3
1–2	257325	41.8
3–4	116684	18.9
≥ 5	49357	8.0

a The sum of the study subjects in each category is smaller than the total study population due to missing values.

b As of 31 Dec 2018.

c Six hospital/clinic visits or receiving a prescription for ≥8 weeks.

d At least one program completion for users with multiple enrollments.

Table 2 shows the medical costs of selected smoking-related diseases during the 1 year before and after the first enrollment in the NHIS smoking cessation service. Among the 779315 service users, 97.2% (n=757510) used medical services for any kind of disease at least once in the year before enrolling in the smoking cessation program. The most prevalent diseases were acute upper respiratory infections (37.0%), followed by acute lower respiratory infections (31.4%), other upper respiratory diseases (20.3%), chronic lower respiratory diseases (12.7%), and diabetes mellitus (10.8%). After cessation service enrollment, the number of patients who accessed care for any kind of disease increased by 1% (n=7487), the number of days spent in the hospital increased by 13%, and medical costs increased by 23%. The number of patients, days spent in the hospital, and medical costs for 1 year consistently increased for all 15 selected diseases after service enrollment. The biggest changes in the number of patients were observed in influenza (64%), cancer (26%), and diseases of the arteries, arterioles, and capillaries (21%). We observed decreased prevalence and medical costs of pneumonia among women and participants aged < 50 years, whereas an increasing pattern was consistently observed for the remaining diseases in both sexes and older age groups (Supplementary file Tables 1 and 2).

**Table 2:** Disease prevalence, length of hospital stay, and medical costs of selected smoking-related diseases for 1 year before and after the first enrollment in the National Health Insurance Service (NHIS) smoking cessation service (N=779315)

*Disease*	*BEFORE service enrollment (1 year)*				*AFTER service enrollment (1 year)*				*Changes (AFTER - BEFORE)*			*Changes (%)^a^*		
	*Number of patients* *n*	*Prevalence* *%*	*Length of hospital stay (days)*	*Medical cost (thousand KRW)*	*Number of patients* *n*	*Prevalence* *%*	*Length of hospital stay (days)*	*Medical cost (thousand KRW)*	*Number of patients* *n*	*Length of hospital stay (days)*	*Medical cost (thousand KRW)*	*Number of patients*	*Length of hospital stay (days)*	*Medical cost*
All^b^	757510	97.2	18808880	1149451124	764997	98.2	21162525	1414704948	7487	2353645	265253824	1	13	23
Tuberculosis	1951	0.3	20444	2820284	2053	0.3	25395	3799350	102	4951	979066	5	24	35
Cancer	16403	2.1	211687	54976840	20594	2.6	396323	115607060	4191	184636	60630220	26	87	110
Diabetes mellitus	84420	10.8	710652	59958666	97121	12.5	859298	75353474	12701	148646	15394808	15	21	26
Cataract	19569	2.5	64780	9801279	22197	2.8	72525	11526856	2628	7745	1725576	13	12	18
Ischemic heart	25280	3.2	130426	48796051	28937	3.7	153399	53188874	3657	22973	4392823	14	18	9
Cerebrovascular	19949	2.6	232688	41068606	22693	2.9	291750	48445206	2744	59062	7376600	14	25	18
Artery arteriole and capillary	9977	1.3	40850	8819758	12071	1.5	50629	11191165	2094	9779	2371407	21	24	27
Acute upper respiratory infections	288370	37.0	740895	16712499	299270	38.4	767119	17940033	10900	26224	1227534	4	4	7
Influenza	7683	1.0	13616	834529	12627	1.6	20879	1303836	4944	7263	469307	64	53	56
Pneumonia	16619	2.1	64169	7274975	16868	2.2	69075	9003410	249	4906	1728434	1	8	24
Acute lower respiratory infections	244470	31.4	622702	15232834	258390	33.2	652718	16497566	13920	30016	1264732	6	5	8
Other upper respiratory	158368	20.3	422634	14914188	175907	22.6	478260	18012478	17539	55626	3098289	11	13	21
Chronic lower respiratory	99085	12.7	348124	20208033	106443	13.7	378409	24001693	7358	30285	3793660	7	9	19
Rheumatoid arthritis	4069	0.5	21889	2807559	4255	0.5	23378	3268613	186	1489	461054	5	7	16
Impotence	1000	0.1	1840	48657	1114	0.1	2084	58876	114	244	10218	11	13	21

a Percent change = [(AFTER-BEFORE)/BEFORE]×100.

b All: ICD-10 codes A00–Z99. KRW: 1100 Korean Won about US$1 (average exchange rate 2015–2017).

**Figure 3:**
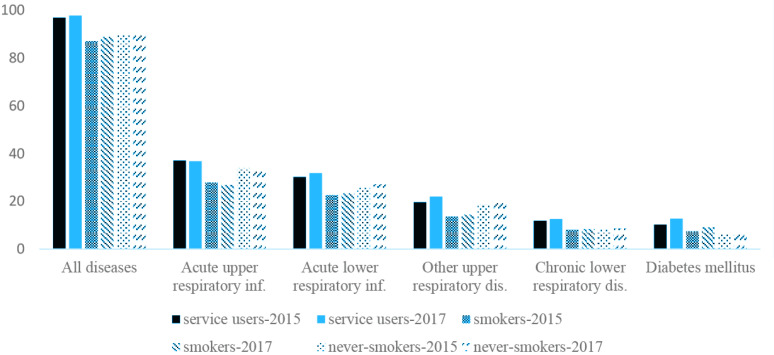
The prevalence of the five most common diseases in 2015 and 2017 among the National Health Insurance Service (NHIS) smoking cessation service users (service users), smokers who did not use the NHIS cessation service (smokers), and never smokers in 2016

Figure 3 and Supplementary file Table 3 show the disease prevalence among cessation service users, smokers who never used the cessation service, and never smokers. Generally, disease prevalence was highest for cessation service users and lowest for smokers who never used the cessation service. For example, the prevalence of all diseases (ICD-10 codes A00–Z99) in 2017 was 97.7%, 88.8%, and 91.1% for cessation service users, smokers who did not use the service, and never smokers, respectively. The prevalence of acute upper respiratory infections in 2017 was highest among cessation service users (36.8%), followed by never smokers (32.9%) and smokers who never used the cessation service (26.8%). Disease prevalence increased more among the cessation service users than among the other groups. For example, the prevalence of ischemic heart diseases increased from 2.9% in 2015 to 3.9% in 2017 (35.6% change) among cessation service users, whereas 20% increases were noted in the other groups. The same pattern was observed for other smoking-related diseases (Supplementary file Table 3-1). However, the number of days spent in the hospital and medical costs for chronic diseases were lowest in never smokers (Supplementary file Tables 3-1 and 3-2).

## DISCUSSION

In the current study, the number of patients, days spent in the hospital, and medical costs for 1 year before and after the use of the NHIS smoking cessation program were measured and compared with those of control groups (smokers who do not use the service and never smokers) to estimate the short-term effect of the cessation service use. From the study results, it was found that the disease-specific prevalence and their medical cost were highest and increased more in the cessation service users compared with the control groups. Particularly, decreased prevalence and medical costs for pneumonia were observed among cessation service users aged <50 years.

Although specific smoking cessation interventions have been suggested to improve the involvement of healthcare providers in cessation support, the interventions are not frequently successful in healthcare settings, and their effectiveness has been evaluated by the cessation success rate or process improvements^19^. Therefore, more studies are needed to measure other outcomes, such as tobacco-related disease prevalence and medical costs, to assess the ultimate goal of cessation interventions: to minimize the health risks and economic burden caused by tobacco use. Furthermore, research on the use of smoking cessation services and healthcare costs using real-world data is necessary to provide a strong context in terms of generalizability, as they are far less.

In these perspectives, a few studies suggested a decrease in some healthcare costs in the year post-smoking cessation treatment, but these findings resulted from the limited study design and target population, such as the comparison of smokers with or without inpatient tobacco dependence treatment during the hospitalization or the comparison of varenicline versus NRT users without comparison to the non-treated group^20,21^. While some previous studies, including two recently published studies from Ontario, Canada, based on the longitudinal follow-up design using real-world measurement of the healthcare cost linked with administrative healthcare data, showed an increase in or relatively higher medical utilization and cost among the smoking cessation service users^22-25^. One study reported higher total healthcare costs in the treatment cohort pre- and post-treatment periods^24^, and another study identified a significantly greater rate of outpatient visits, emergency department visits and hospitalizations among patients who enrolled in smoking cessation treatment offered through primary care clinics over a 5-year follow-up period^25^. The above suggested that the increase, which contradicts other prior studies’ results, may be explained by more access to medical care for smoking cessation service users for their various health concerns or differences in treatment settings, patient populations, and healthcare service outcome measures among the studies.

Our findings correspond to the two recent publications and have some strong points in the data used, study design applied, and evidence introduced. The current study is based on the NHIS claims data, which include comprehensive information on disease code, medical cost for each inpatient and outpatient service or care, and other information on socioeconomic and medical status with individual base linkage to the data from NHIS general health screening participants. Moreover, NHIS claims data are highly representative of the Korean population, as they cover the entire population in Korea. The change in disease prevalence and medical cost were compared with smokers who do not use the smoking cessation service and never smokers, as well as within smoking cessation service users in the 1-year period before and after service enrollment. Known smoking-related diseases are classified and introduced for each comparison on the change in medical cost, which could give information on the different short-term effects of smoking cessation service use by disease-specific characteristics.

The long-term health benefits of smoking cessation are well known. Cessation reduces mortality, carrying respective gains of approximately 3, 6, 9, and 10 years of life expectancy for those aged 30–60 years^26^. The short-term economic and health benefits of smoking cessation have also been demonstrated for acute myocardial infarction (AMI) and stroke, in modelling studies using US^27^ and Australian data^28^. In the Australian simulation, if smoking prevalence had decreased by 1% in the first year, approximately 1000 hospitalizations for AMI and 350 for stroke would have been avoided over 7 years, saving approximately $20.4 million in healthcare costs^28^. However, our observational study based on real data showed increased medical costs and the prevalence of diseases post-cessation service use. This was true even for acute respiratory infections, which can be considered a target disease for measuring short-term health benefits. Our results are comparable with those of other observational studies. Previous research on the healthcare costs associated with former smokers has suggested that those who quit smoking may incur greater healthcare costs than continuing smokers. For example, in a cross-sectional study in Germany, the probability of hospitalization was highest among those who quit 1–3 years ago and decreased thereafter^29^. In a retrospective cohort study of enrollees in a health maintenance organization in the US, former smokers’ costs were significantly greater in the year immediately following cessation relative to those incurred by continuing smokers^22^. More specifically, the healthcare costs among former smokers began to rise in the quarter prior to cessation and were significantly greater than those of continuing smokers in the quarter immediately following cessation^23^. However, former smokers’ costs fell in year two, and this decrease was maintained throughout the 6-year follow-up period^22^. Increased hospital utilization in the first year after quitting smoking is more likely to be a cause of smoking cessation than a consequence. Specifically, short-term excess healthcare utilization among former smokers may result from smoking-related conditions that led to smoking cessation^29^. After quitting, former smokers may seek medical care that they delayed while they were smokers, resulting in increased healthcare utilization^30^. On the other hand, in a randomized controlled trial of pharmacotherapy and counselling for smoking cessation, sustained quitters amassed lower healthcare costs by the sixth quarter post-quitting than trial participants who continued smoking^31^.

The results of the present study help us to understand the characteristics of smokers who use the cessation program. Among the cessation service users, the prevalence of smoking-related diseases and their medical costs increased in the year following service enrollment. Notably, the disease prevalence and medical costs for pneumonia were lowest among individuals aged <50 years. This finding may provide evidence that the short-term benefits of the cessation program are greater for younger smokers than for older adult smokers. On the other hand, compared with matched control groups by age, sex, income, and residential area, the prevalence of most of the selected diseases was highest among the cessation service users. For example, the prevalence of acute upper respiratory infections in 2017 was 36.8%, 32.9%, and 26.8% in the groups of cessation service users, never smokers, and smokers who never used the cessation service, respectively. As another example of chronic disease, the prevalence of diabetes mellitus in 2017 was 12.8%, 9.1%, and 7.7% in the groups of cessation service users, smokers who never used the cessation service, and never smokers, respectively. We also observed increased prevalence and medical costs between 2015 and 2017 for most diseases in all three groups, though the increase was highest among the cessation service users. This finding may be explained by cessation service users being more vulnerable to diseases and the cessation program simply targeting the right people. Interestingly, for most of the selected diseases, the smokers who never used the NHIS cessation service showed a lower disease prevalence than both service users and never smokers. Moreover, in our results, healthcare utilization for any kind of disease was highest among cessation service users and lowest among smokers who did not use the service. One plausible explanation is that smokers who need medical services may be motivated to use the cessation service, whereas the remaining smokers are less likely to need and seek healthcare. Thus, smokers who never use the cessation service are healthy enough to continue indulging in their smoking habit. Quitters may seek medical care that they delayed while they were smokers, resulting in increased healthcare utilization upon service enrollment^29,30^.

### Limitations

This study has several limitations. First, we investigated the changes in disease prevalence and related medical costs among the NHIS cessation service users, not among quitters. Fewer than half of the service users successfully completed the program by attending six hospital/clinic visits or receiving a prescription for 56 days (20.6%, 40.1%, and 44.3% in 2015, 2016, and 2017, respectively). More than half of enrollees dropped out after using the service one or two times^14^. According to a self-reported telephone survey, the continuous 6-month abstinence rate among NHIS cessation service users was 30.5%^15^. Therefore, the effect of NHIS cessation service use might be heterogenous by the duration smokers engaged in the cessation program, and the high proportion of drop-out could impact the estimation of the overall amount of medical cost as well as disease prevalence. Second, this study observed changes in disease prevalence and medical costs for one year after service enrollment. However, this period is too short to observe the clear clinical and economic benefits of the cessation service. Previous studies estimated the clinical and economic benefits of smoking cessation for up to 7 years as ‘short-term’^30^. Hospital use for quitters increases in the short-term but declines over the subsequent 4–10 years^29^. Further studies must include a longer follow-up period for clinical and economic changes, including acute and chronic diseases, among both cessation service users and successful quitters. Third, this study observed disease prevalence, not incidence, and we were unable to evaluate the effect of cessation service use on primary prevention (i.e. prevention before health problems occur). Lastly, disease prevalence was based on primary diagnosis; secondary and other sub-diagnoses were excluded, which may result in underestimation when considering co-morbidity. However, the potential risk of underestimation was likely non-differential in each group being compared.

Despite these limitations, our study results add evidence of the increase in medical costs as well as smoking related disease prevalence, although it is not consistent for all smoking related diseases by gender and age group, with the assessment of the real-world effectiveness of national smoking cessation interventions in healthcare settings requiring a longitudinal follow-up design in a large population.

## CONCLUSIONS

Cessation service users were more likely to seek healthcare. Increased healthcare utilization in the first year after cessation service use may have resulted from smoking-related conditions that led individuals to attempt smoking cessation.

## Data Availability

The data supporting this research cannot be made available for privacy or other reasons.
