# Supplementary material for: Short-term effects of national smoking cessation service on smoking-related disease prevalence and healthcare costs: Experience from the National Health Insurance Service Smoking Cessation Intervention Program in Korea
Source: Tob Induc Dis. 2023 Aug 24;21:107. doi: 10.18332/tid/169654 (PMC10448735; doi:10.18332/tid/169654)
Supplement: Supplementary file 1 [file TID-21-107-s1.pdf]

Supplementary Table 1-1. Disease prevalence, length of hospital stays, and medical costs of selected smoking-related diseases for 1 year before and after the first enrollment in the National Health Insurance Service (NHIS) smoking cessation service among men (N=673,967)

Disease	BEFORE service enrollment (1 year)				AFTER service enrollment (1 year)				Change (AFTER−BEFORE)			Change (%) [a]		
	Number of patients	Prevalence (%)	Length of hospital stay (days)	Medical cost (thousand Korean won)	Number of patients	Prevalence (%)	Length of hospital stay (days)	Medical cost (thousand Korean won)	Number of patients	Length of hospital stay (days)	Medical cost (thousand Korean won)	Number of patients	Length of hospital stay (days)	Medical cost
Total [b]	653,351	96.9	15,202,450	954,924,943	660,369	98.0	17,221,638	1,181,348,858	7,018	2,019,188	226,423,915	1	13	24
Tuberculosis	1,762	0.3	19,096	2,638,883	1,888	0.3	23,913	3,590,852	126	4,817	951,969	7	25	36
Cancer	13,692	2.0	176,274	47,408,976	17,356	2.6	340,250	101,787,789	3,664	163,976	54,378,813	27	93	115
Diabetes mellitus	74,961	11.1	622,098	52,618,321	86,291	12.8	754,881	66,359,494	11,330	132,783	13,741,174	15	21	26
Cataracts	16,561	2.5	54,419	8,191,333	18,705	2.8	60,628	9,591,238	2,144	6,209	1,399,906	13	11	17
Ischemic heart dis.	23,071	3.4	117,477	45,844,345	26,373	3.9	138,582	49,707,793	3,302	21,105	3,863,448	14	18	8
Cerebrovascular dis.	17,412	2.6	204,297	35,750,302	19,782	2.9	258,174	42,369,417	2,370	53,877	6,619,115	14	26	19
Artery, arteriole, and capillary dis.	8,347	1.2	35,888	8,168,872	10,050	1.5	43,345	10,059,477	1,703	7,457	1,890,606	20	21	23
Acute upper respiratory inf.	239,985	35.6	597,818	13,428,470	250,092	37.1	625,066	14,570,126	10,107	27,248	1,141,655	4	5	9
Influenza	6,343	0.9	10,805	628,976	10,501	1.6	16,889	1,045,824	4,158	6,084	416,848	66	56	66
Pneumonia	13,232	2.0	49,803	5,890,855	13,712	2.0	55,850	7,597,205	480	6,047	1,706,351	4	12	29
Acute lower respiratory inf.	200,922	29.8	489,573	11,876,904	214,263	31.8	521,988	13,099,452	13,341	32,415	1,222,548	7	7	10
Other upper respiratory dis.	130,997	19.4	346,462	12,489,947	146,452	21.7	396,735	15,222,666	15,455	50,273	2,732,719	12	15	22
Chronic lower respiratory dis.	80,342	11.9	272,441	16,108,568	87,279	13.0	301,156	19,513,477	6,937	28,715	3,404,909	9	11	21
Rheumatoid arthritis	2,698	0.4	13,235	1,780,096	2,902	0.4	14,918	2,093,563	204	1,683	313,466	8	13	18
Impotence	1,000	0.1	1,840	48,657	1114	0.2	2,084	58,876	114	244	10,218	11	13	21

[a] Change (%): change (AFTER−BEFORE) ÷ BEFORE × 100; [b] All: ICD-10 codes A00–Z99; 1 USD ≒ 1,100 Korean won (average exchange rate during 2015–2017)

Supplementary Table 1-2. Disease prevalence, length of hospital stays, and medical costs of selected smoking-related diseases for 1 year before and after the first enrollment in the National Health Insurance Service (NHIS) smoking cessation service among women (N=105,348)

Disease	BEFORE service enrollment (1 year)				AFTER service enrollment (1 year)				Change (AFTER−BEFORE)			Change (%) [a]		
	Number of patients	Prevalence (%)	Length of hospital stay (days)	Medical cost (thousand Korean won)	Number of patients	Prevalence (%)	Length of hospital stay (days)	Medical cost (thousand Korean won)	Number of patients	Length of hospital stay (days)	Medical cost (thousand Korean won)	Number of patients	Length of hospital stay (days)	Medical cost
All [b]	104,159	98.9	3,606,430	194,526,181	104,628	99.3	3,940,887	233,356,090	469	334,457	38,829,909	0	9	20
Tuberculosis	189	0.2	1,348	181,402	165	0.2	1,482	208,499	−24	134	27,097	−13	10	15
Cancer	2,711	2.6	35,413	7,567,864	3,238	3.1	56,073	13,819,271	527	20,660	6,251,407	19	58	83
Diabetes mellitus	9,459	9.0	88,554	7,340,346	10,830	10.3	104,417	8,993,980	1,371	15,863	1,653,634	14	18	23
Cataracts	3,008	2.9	10,361	1,609,947	3,492	3.3	11,897	1,935,617	484	1,536	325,670	16	15	20
Ischemic heart dis.	2,209	2.1	12,949	2,951,706	2,564	2.4	14,817	3,481,081	355	1,868	529,375	16	14	18
Cerebrovascular dis.	2,537	2.4	28,391	5,318,303	2,911	2.8	33,576	6,075,788	374	5,185	757,485	15	18	14
Artery, arteriole, and capillary dis.	1,630	1.5	4,962	650,886	2,021	1.9	7,284	1,131,687	391	2,322	480,801	24	47	74
Acute upper respiratory inf.	48,385	45.9	143,077	3,284,029	49,178	46.7	142,053	3,369,907	793	−1,024	85,878	2	−1	3
Influenza	1,340	1.3	2,811	205,553	2,126	2.0	3,990	258,012	786	1,179	52,459	59	42	26
Pneumonia	3,387	3.2	14,366	1,384,121	3,156	3.0	13,225	1,406,204	−231	−1,141	22,083	−7	−8	2
Acute lower respiratory inf.	43,548	41.3	133,129	3,355,930	44,127	41.9	130,730	3,398,113	579	−2,399	42,183	1	−2	1
Other upper respiratory dis.	27,371	26.0	76,172	2,424,241	29,455	28.0	81,525	2,789,811	2,084	5,353	365,570	8	7	15
Chronic lower respiratory dis.	18,743	17.8	75,683	4,099,465	19,164	18.2	77,253	4,488,215	421	1,570	388,750	2	2	9
Rheumatoid arthritis	1,371	1.3	8,654	1,027,463	1,353	1.3	8,460	1,175,051	−18	−194	147,588	−1	−2	14

[a] Change (%): change (AFTER−BEFORE) ÷ BEFORE × 100; [b] All: ICD-10 codes A00–Z99; 1 USD ≒ 1,100 Korean won (average exchange rate during 2015–2017)

Supplementary Table 2-1. Disease prevalence, length of hospital stays, and medical costs of selected smoking-related diseases for 1 year before and after the first enrollment in the National Health Insurance Service (NHIS) smoking cessation service among 19–39-year-olds (N=199,897)

Disease	BEFORE service enrollment (1 year)				AFTER service enrollment (1 year)				Change (AFTER−BEFORE)			Change (%) [a]		
	Number of patients	Preva-lence (%)	Length of hospital stay (days)	Medical cost (thousand Korean won)	Number of patients	Preva-lence (%)	Length of hospital stay (days)	Medical cost (thousand Korean won)	Number of patients	Length of hospital stay (days)	Medical cost (thousand Korean won)	Number of patients	Length of hospital stay (days)	Medical cost
All [b]	190,985	95.5	2,906,668	151,163,550	193,680	96.9	3,229,769	174,935,942	2,695	323,101	23,772,391	1	11	16
Tuberculosis	272	0.1	2,882	339,336	253	0.1	2,516	370,113	−19	−366	30,777	−7	−13	9
Cancer	907	0.5	9,626	2,318,366	1,103	0.6	14,399	4,150,439	196	4,773	1,832,073	22	50	79
Diabetes mellitus	3,844	1.9	26,873	2,277,515	4,993	2.5	37,688	3,298,184	1,149	10,815	1,020,668	30	40	45
Cataracts	208	0.1	548	93,230	242	0.1	628	99,795	34	80	6,566	16	15	7
Ischemic heart dis.	740	0.4	2,856	1,139,512	905	0.5	3,534	1,182,801	165	678	43,289	22	24	4
Cerebrovascular dis.	558	0.3	7,412	1,683,948	666	0.3	7,764	1,496,720	108	352	−187,228	19	5	−11
Artery, arteriole, and capillary dis.	543	0.3	1,766	239,760	621	0.3	2,146	401,553	78	380	161,793	14	22	67
Acute upper respiratory inf.	81,984	41.0	195,267	4,657,664	85,756	42.9	206,445	5,105,167	3,772	11,178	447,503	5	6	10
Influenza	2,696	1.3	4,360	242,387	4,139	2.1	6,333	351,058	1,443	1,973	108,671	54	45	45
Pneumonia	3,083	1.5	8,159	710,167	2,952	1.5	7,153	563,716	−131	−1,006	−146,451	−4	−12	−21
Acute lower respiratory inf.	64,295	32.2	141,820	3,444,245	68,822	34.4	152,773	3,802,902	4,527	10,953	358,657	7	8	10
Other upper respiratory dis.	46,237	23.1	111,711	4,091,227	51,141	25.6	126,113	4,960,505	4,904	14,402	869,278	11	13	21
Chronic lower respiratory dis.	19,041	9.5	42,374	1,802,429	20,426	10.2	45,341	1,984,897	1,385	2,967	182,468	7	7	10
Rheumatoid arthritis	415	0.2	1,401	193,768	430	0.2	1,432	184,251	15	31	−9,517	4	2	−5
Impotence	204	0.1	389	8,542	229	0.1	503	9,948	25	114	1,406	12	29	16

[a] Change (%): change (AFTER−BEFORE) ÷ BEFORE × 100; [b] All: ICD-10 codes A00–Z99; 1 USD ≒ 1,100 Korean won (average exchange rate during 2015–2017)

Supplementary Table 2-2. Disease prevalence, length of hospital stays, and medical costs of selected smoking-related diseases for 1 year before and after the first enrollment in the National Health Insurance Service (NHIS) smoking cessation service among 40–49-year-olds (N=230,801)

Disease	BEFORE service enrollment (1 year)				AFTER service enrollment (1 year)				Change (AFTER−BEFORE)			Change (%) [a]		
	Number of patients	Prevalence (%)	Length of hospital stay (days)	Medical cost (thousand Korean won)	Number of patients	Prevalence (%)	Length of hospital stay (days)	Medical cost (thousand Korean won)	Number of patients	Length of hospital stay (days)	Medical cost (thousand Korean won)	Number of patients	Length of hospital stay (days)	Medical cost
All [b]	223,048	96.6	4,460,380	255,257,626	225,731	97.8	5,010,895	301,473,711	2,683	550,515	46,216,085	1	12	18
Tuberculosis	463	0.2	5,704	823,357	462	0.2	6,450	828,913	−1	746	5,556	0	13	1
Cancer	2,663	1.2	30,867	7,615,294	3,259	1.4	56,069	15,310,457	596	25,202	7,695,162	22	82	101
Diabetes mellitus	17,043	7.4	133,199	11,154,545	20,665	9.0	171,841	14,970,133	3,622	38,642	3,815,589	21	29	34
Cataracts	1,443	0.6	4,231	761,123	1,788	0.8	5,267	973,558	345	1,036	212,434	24	24	28
Ischemic heart dis.	3,353	1.5	16,811	7,434,357	4,037	1.7	20,784	7,127,260	684	3,973	−307,097	20	24	−4
Cerebrovascular dis.	2,415	1.0	33,828	6,560,344	2,854	1.2	41,674	7,365,375	439	7,846	805,030	18	23	12
Artery, arteriole, and capillary dis.	1,427	0.6	5,625	818,944	1,830	0.8	7,071	1,215,814	403	1,446	396,870	28	26	48
Acute upper respiratory inf.	84,077	36.4	206,577	4,742,313	87,375	37.9	214,864	5,062,821	3,298	8,287	320,508	4	4	7
Influenza	2,407	1.0	3,983	211,863	3,989	1.7	6,181	338,897	1,582	2,198	127,034	66	55	60
Pneumonia	3,754	1.6	11,259	1,093,768	3,535	1.5	10,324	1,081,579	−219	−935	−12,189	−6	−8	−1
Acute lower respiratory inf.	69,334	30.0	163,431	3,945,893	73,187	31.7	169,870	4,233,435	3,853	6,439	287,541	6	4	7
Other upper respiratory dis.	45,833	19.9	117,459	4,212,827	50,875	22.0	132,649	5,008,837	5,042	15,190	796,010	11	13	19
Chronic lower respiratory dis.	21,674	9.4	55,644	2,622,138	23,356	10.1	59,439	2,897,496	1,682	3,795	275,359	8	7	11
Rheumatoid arthritis	813	0.4	4,050	468,782	904	0.4	4,235	507,957	91	185	39,175	11	5	8
Impotence	230	0.1	397	11,788	241	0.1	396	11,053	11	−1	−735	5	0	−6

[a] Change (%): change (AFTER−BEFORE) ÷ BEFORE × 100; [b] All: ICD-10 codes A00–Z99; 1 USD ≒ 1,100 Korean won (average exchange rate during 2015–2017)

Supplementary Table 2-3. Disease prevalence, length of hospital stays, and medical costs of selected smoking-related diseases for 1 year before and after the first enrollment in the National Health Insurance Service (NHIS) smoking cessation service among 50–59-year-olds (N=205,898)

Disease	BEFORE service enrollment (1 year)				AFTER service enrollment (1 year)				Change (AFTER−BEFORE)			Change (%) [a]		
	Number of patients	Prevalence (%)	Length of hospital stay (days)	Medical cost (thousand Korean won)	Number of patients	Prevalence (%)	Length of hospital stay (days)	Medical cost (thousand Korean won)	Number of patients	Length of hospital stay (days)	Medical cost (thousand Korean won)	Number of patients	Length of hospital stay (days)	Medical cost
All [b]	201,876	98.0	5,658,229	360,489,073	203,480	98.8	6,337,921	435,526,324	1,604	679,692	75,037,251	1	12	21
Tuberculosis	627	0.3	6,772	969,149	613	0.3	8,686	1,290,700	−14	1,914	321,551	−2	28	33
Cancer	5,181	2.5	74,242	19,218,892	6,428	3.1	129,866	37,440,818	1,247	55,624	18,221,926	24	75	95
Diabetes mellitus	31,210	15.2	262,227	22,424,146	35,805	17.4	315,668	28,141,299	4,595	53,441	5,717,154	15	20	25
Cataracts	4,761	2.3	15,180	2,460,845	5,851	2.8	17,708	3,198,935	1,090	2,528	738,090	23	17	30
Ischemic heart dis.	8,484	4.1	44,482	17,642,420	9,857	4.8	51,726	18,255,129	1,373	7,244	612,709	16	16	3
Cerebrovascular dis.	6,534	3.2	87,387	14,904,579	7,412	3.6	103,403	17,119,642	878	16,016	2,215,063	13	18	15
Artery, arteriole, and capillary dis.	3,108	1.5	12,058	2,304,139	3,783	1.8	14,826	2,677,544	675	2,768	373,406	22	23	16
Acute upper respiratory inf.	69,188	33.6	176,465	3,932,813	71,801	34.9	181,475	4,201,798	2,613	5,010	268,985	4	3	7
Influenza	1,560	0.8	2,957	188,834	2,837	1.4	4,975	327,808	1,277	2,018	138,973	82	68	74
Pneumonia	4,211	2.0	17,358	2,052,000	4,316	2.1	17,785	2,220,467	105	427	168,467	2	2	8
Acute lower respiratory inf.	59,800	29.0	152,882	3,816,148	63,693	30.9	161,448	4,166,113	3,893	8,566	349,965	7	6	9
Other upper respiratory dis.	37,167	18.1	102,205	3,749,443	41,930	20.4	117,756	4,588,762	4,763	15,551	839,319	13	15	22
Chronic lower respiratory dis.	25,508	12.4	84,382	4,959,551	27,739	13.5	92,960	5,835,000	2,231	8,578	875,449	9	10	18
Rheumatoid arthritis	1,403	0.7	7,617	943,244	1,469	0.7	8,452	1,175,558	66	835	232,314	5	11	25
Impotence	292	0.1	511	13,524	351	0.2	628	19,958	59	117	6,435	20	23	48

[a] Change (%): change (AFTER−BEFORE) ÷ BEFORE × 100; [b] All: ICD-10 codes A00–Z99; 1 USD ≒ 1,100 Korean won (average exchange rate during 2015–2017)

Supplementary Table 2-4. Disease prevalence, length of hospital stays, and medical costs of selected smoking-related diseases for 1 year before and after the first enrollment in the National Health Insurance Service (NHIS) smoking cessation service among those over 60 years of age (N=142,719)

Disease	BEFORE service enrollment (1 year)				AFTER service enrollment (1 year)				Change (AFTER−BEFORE)			Change (%) [a]		
	Number of patients	Prevalence (%)	Length of hospital stay (days)	Medical cost (thousand Korean won)	Number of patients	Prevalence (%)	Length of hospital stay (days)	Medical cost (thousand Korean won)	Number of patients	Length of hospital stay (days)	Medical cost (thousand Korean won)	Number of patients	Length of hospital stay (days)	Medical cost
All [b]	141,601	99.2	5,783,603	382,540,876	142,106	99.6	6,583,940	502,768,972	505	800,337	120,228,096	0	14	31
Tuberculosis	589	0.4	5,086	688,337	725	0.5	7,743	1,309,624	136	2,657	621,287	23	52	90
Cancer	7,652	5.4	96,952	25,824,287	9,804	6.9	195,989	58,705,347	2,152	99,037	32,881,060	28	102	127
Diabetes mellitus	32,323	22.6	288,353	24,102,460	35,658	25.0	334,101	28,943,857	3,335	45,748	4,841,397	10	16	20
Cataracts	13,157	9.2	44,821	6,486,082	14,316	10.0	48,922	7,254,567	1,159	4,101	768,486	9	9	12
Ischemic heart dis.	12,703	8.9	66,277	22,579,762	14,138	9.9	77,355	26,623,684	1,435	11,078	4,043,922	11	17	18
Cerebrovascular dis.	10,442	7.3	104,061	17,919,734	11,761	8.2	138,909	22,463,469	1,319	34,848	4,543,735	13	33	25
Artery, arteriole, and capillary dis.	4,899	3.4	21,401	5,456,916	5,837	4.1	26,586	6,896,253	938	5,185	1,439,338	19	24	26
Acute upper respiratory inf.	53,121	37.2	162,586	3,379,708	54,338	38.1	164,335	3,570,247	1,217	1,749	190,538	2	1	6
Influenza	1,020	0.7	2,316	191,444	1,662	1.2	3,390	286,073	642	1,074	94,629	63	46	49
Pneumonia	5,571	3.9	27,393	3,419,040	6,065	4.2	33,813	5,137,647	494	6,420	1,718,607	9	23	50
Acute lower respiratory inf.	51,041	35.8	164,569	4,026,548	52,688	36.9	168,627	4,295,116	1,647	4,058	268,568	3	2	7
Other upper respiratory dis.	29,131	20.4	91,259	2,860,691	31,961	22.4	101,742	3,454,373	2,830	10,483	593,682	10	11	21
Chronic lower respiratory dis.	32,862	23.0	165,724	10,823,915	34,922	24.5	180,669	13,284,299	2,060	14,945	2,460,385	6	9	23
Rheumatoid arthritis	1,438	1.0	8,821	1,201,594	1,452	1.0	9,259	1,400,848	14	438	199,254	1	5	17
Impotence	274	0.2	543	14,562	293	0.2	557	17,916	19	14	3,354	7	3	23

[a] Change (%): change (AFTER−BEFORE) ÷ BEFORE × 100; [b] All: ICD-10 codes A00–Z99; 1 USD ≒ 1,100 Korean won (average exchange rate during 2015–2017)

Supplementary Table 3-1. Changes in the **number of patients** in 2015 and 2017 among National Health Insurance Service (NHIS) smoking cessation service users enrolled in 2016, smokers who did not use the NHIS smoking cessation service, and never smokers

	NHIS cessation service users (N=231,204)					Smokers who never used the NHIS cessation service (N=231,204)					Never smokers (N=231,204)				
Disease	No. of patients in 2015	Prevalence (%)	No. of patients in 2017	Prevalence (%)	Change (%)[a]	No. of patients in 2015	Prevalence (%)	No. of patients in 2017	Prevalence (%)	Change (%)[a]	No. of patients in 2015	Prevalence (%)	No. of patients in 2017	Prevalence (%)	Change (%)[a]
All [b]	223,859	96.8	225,831	97.7	0.9	201,059	87.0	205,284	88.8	2.1	207,446	89.7	210,701	91.1	0.6
Tuberculosis	512	0.2	547	0.2	6.8	441	0.2	462	0.2	4.8	378	0.2	342	0.1	−9.5
Cancer	4,250	1.8	6,822	3.0	60.5	2,916	1.3	4,274	1.8	46.6	4,974	2.2	6,155	2.7	23.7
Diabetes mellitus	23,527	10.2	29,485	12.8	25.3	17,275	7.5	21,023	9.1	21.7	14,758	6.4	17,717	7.7	20.1
Cataracts	5,550	2.4	6,991	3.0	26.0	4,071	1.8	4,994	2.2	22.7	5,140	2.2	6,424	2.8	25.0
Ischemic heart dis.	6,729	2.9	9,122	3.9	35.6	4,248	1.8	5,084	2.2	19.7	4,119	1.8	4,943	2.1	20.0
Cerebrovascular dis.	4,933	2.1	6,717	2.9	36.2	3,405	1.5	4,268	1.8	25.3	3,917	1.7	4,531	2.0	15.7
Artery, arteriole, and capillary dis.	2,594	1.1	3,514	1.5	35.5	1,558	0.7	1,974	0.9	26.7	1,446	0.6	1,926	0.8	33.2
Acute upper respiratory inf.	85,550	37.0	85,125	36.8	−0.5	64,239	27.8	61,855	26.8	−3.7	78,446	33.9	76,166	32.9	−2.9
Influenza	1,962	0.8	2,632	1.1	34.1	1,396	0.6	2,176	0.9	55.9	1,889	0.8	2,672	1.2	41.5
Pneumonia	4,523	2.0	4,496	1.9	−0.6	2,942	1.3	3,009	1.3	2.3	3,377	1.5	3,284	1.4	−2.8
Acute lower respiratory inf.	69,894	30.2	73,520	31.8	5.2	52,145	22.6	53,923	23.3	3.4	59,274	25.6	62,680	27.1	5.7
Other upper respiratory dis.	45,302	19.6	50,632	21.9	11.8	31,490	13.6	33,110	14.3	5.1	42,930	18.6	45,143	19.5	5.2
Chronic lower respiratory dis.	27,408	11.9	29,074	12.6	6.1	18,399	8.0	19,198	8.3	4.3	19,488	8.4	20,093	8.7	3.1
Rheumatoid arthritis	1,236	0.5	1,250	0.5	1.1	839	0.4	882	0.4	5.1	940	0.4	883	0.4	−6.1
Impotence	282	0.1	292	0.1	3.5	153	0.1	183	0.1	19.6	165	0.1	187	0.1	13.3

[a] Change (%): change (2017−2015) ÷ 2015 × 100; [b] All: ICD-10 codes A00–Z99

Supplementary Table 3-2. Changes in the **number of days spent in the hospital** in 2015 and 2017 among National Health Insurance Service (NHIS) smoking cessation service users enrolled in 2016, smokers who did not use the NHIS cessation service, and never smokers

Disease	NHIS cessation service users			Smokers who never used the NHIS cessation service			Never smokers		
	Days spent in the hospital		Change (%)[a]	Days spent in the hospital		Change (%)[a]	Days spent in the hospital		Change (%)[a]
	2015	2017		2015	2017		2015	2017	
All [b]	5,173,022	6,028,142	16.5	3,070,521	3,449,750	12.4	3,383,111	3,725,213	10.1
Tuberculosis	3,956	6,358	60.7	4,064	5,020	23.5	4,139	3,005	−27.4
Cancer	51,651	139,292	169.7	37,715	83,287	120.8	52,022	81,362	56.4
Diabetes mellitus	195,466	251,173	28.5	135,482	165,170	21.9	112,899	135,090	19.7
Cataracts	18,228	22,926	25.8	13,616	16,282	19.6	16,707	20,031	19.9
Ischemic heart dis.	33,677	46,223	37.3	20,773	24,922	20.0	18,442	21,873	18.6
Cerebrovascular dis.	48,043	80,903	68.4	47,669	65,317	37.0	60,816	64,311	5.7
Artery, arteriole, and capillary dis.	9,893	14,972	51.3	5,838	7,487	28.2	4,405	6,041	37.1
Acute upper respiratory inf.	215,716	213,718	−0.9	146,721	138,513	−5.6	195,377	186,563	−4.5
Influenza	3,820	4,346	13.8	2,545	3,347	31.5	3,667	4,308	17.5
Pneumonia	16,522	19,416	17.5	10,904	13,279	21.8	12,465	14,990	20.3
Acute lower respiratory inf.	173,955	182,062	4.7	115,888	121,860	5.2	139,402	148,133	6.3
Other upper respiratory dis.	118,001	135,294	14.7	74,999	78,358	4.5	110,946	116,066	4.6
Chronic lower respiratory dis.	91,432	101,491	11.0	52,375	56,934	8.7	51,607	54,944	6.5
Rheumatoid arthritis	6,390	7,064	10.5	5,055	5,459	8.0	4,825	4,570	−5.3
Impotence	477	490	2.7	258	331	28.3	302	299	−1.0

[a] Change (%): change (2017−2015) ÷ 2015 × 100; [b] All: ICD-10 codes A00–Z99.

Supplementary Table 3-3. Changes in **medical costs** in 2015 and 2017 among National Health Insurance Service (NHIS) smoking cessation service users enrolled in 2016, smokers who did not use the NHIS cessation service, and never smokers

Disease	NHIS cessation service users			Smokers who never used the NHIS cessation service			Never smokers		
	Medical cost (thousand won)		Change (%)[a]	Medical cost (thousand won)		Change (%)[a]	Medical cost (thousand won)		Change (%)[a]
	2015	2017		2015	2017		2015	2017	
All [b]	289,261,259	426,139,201	47.3	195,718,724	270,125,470	38.0	209,913,799	274,510,579	30.8
Tuberculosis	494,249	1,089,832	120.5	475,524	835,574	75.7	524,423	526,790	0.5
Cancer	12,362,433	41,732,040	237.6	9,767,806	26,121,488	167.4	13,504,132	25,565,856	89.3
Diabetes mellitus	15,691,136	23,102,094	47.2	11,096,255	15,453,224	39.3	8,944,417	12,291,469	37.4
Cataracts	2,692,543	3,688,741	37.0	2,105,959	2,693,549	27.9	2,182,497	2,829,469	29.6
Ischemic heart dis.	10,153,359	16,203,025	59.6	5,867,978	9,019,729	53.7	5,306,924	7,677,727	44.7
Cerebrovascular dis.	7,530,244	14,577,300	93.6	7,136,729	11,264,506	57.8	7,971,386	10,040,726	26.0
Artery, arteriole, and capillary dis.	1,793,082	3,384,518	88.8	1,070,272	1,749,586	63.5	755,600	1,323,515	75.2
Acute upper respiratory inf.	4,682,256	5,089,473	8.7	3,171,221	3,305,432	4.2	4,139,695	4,268,922	3.1
Influenza	231,321	316,791	36.9	152,916	228,023	49.1	226,761	287,994	27.0
Pneumonia	1,651,072	2,738,438	65.9	1,131,248	1,832,260	62.0	1,270,459	2,021,100	59.1
Acute lower respiratory inf.	4,184,992	4,677,214	11.8	2,724,936	3,103,554	13.9	3,201,468	3,667,841	14.6
Other upper respiratory dis.	3,976,328	5,190,908	30.5	2,517,299	2,901,053	15.2	3,445,139	3,978,096	15.5
Chronic lower respiratory dis.	4,848,233	6,754,074	39.3	2,468,393	3,346,062	35.6	2,306,118	2,834,229	22.9
Rheumatoid arthritis	736,202	1,161,392	57.8	654,814	748,254	14.3	624,579	672,963	7.7
Impotence	11,508	14,389	25.0	6,934	9,220	33.0	9,238	11,141	20.6

[a] Change (%): change (2017−2015) ÷ 2015 × 100; [b] All: ICD-10 codes A00–Z99; 1 USD ≒ 1,100 Korean won (average exchange rate during 2015–2017)

© 2023 Oh J.K. et al.
